# Morphogene‐assisted transformation of *Sorghum bicolor* allows more efficient genome editing

**DOI:** 10.1111/pbi.13754

**Published:** 2021-12-16

**Authors:** Kiflom Aregawi, Jianqiang Shen, Grady Pierroz, Manoj K. Sharma, Jeffery Dahlberg, Judith Owiti, Peggy G. Lemaux

**Affiliations:** ^1^ Department of Plant and Microbial Biology University of California Berkeley CA USA; ^2^ University of California Ag & Natural Resources Kearney Agricultural Research & Extension Center Parlier CA USA

**Keywords:** Sorghum, Agrobacterium, engineering, CRISPR/Cas9 editing, morphogene‐assisted transformation, altruistic morphogene‐assisted transformation

## Abstract

*Sorghum bicolor* (L.) Moench, the fifth most important cereal worldwide, is a multi‐use crop for feed, food, forage and fuel. To enhance the sorghum and other important crop plants, establishing gene function is essential for their improvement. For sorghum, identifying genes associated with its notable abiotic stress tolerances requires a detailed molecular understanding of the genes associated with those traits. The limits of this knowledge became evident from our earlier in‐depth sorghum transcriptome study showing that over 40% of its transcriptome had not been annotated. Here, we describe a full spectrum of tools to engineer, edit, annotate and characterize sorghum’s genes. Efforts to develop those tools began with a morphogene‐assisted transformation (MAT) method that led to accelerated transformation times, nearly half the time required with classical callus‐based, non‐MAT approaches. These efforts also led to expanded numbers of amenable genotypes, including several not previously transformed or historically recalcitrant. Another transformation advance, termed altruistic, involved introducing a gene of interest in a separate Agrobacterium strain from the one with morphogenes, leading to plants with the gene of interest but without morphogenes. The MAT approach was also successfully used to edit a target exemplary gene, phytoene desaturase. To identify single‐copy transformed plants, we adapted a high‐throughput technique and also developed a novel method to determine transgene independent integration. These efforts led to an efficient method to determine gene function, expediting research in numerous genotypes of this widely grown, multi‐use crop.

## Introduction

For many crops, efficient transformation methods (hereafter referring to both engineering and editing) are not available. An example is sorghum [*Sorghum bicolor* (L.) Moench], a cereal crop with multiple abiotic stress tolerances (Paterson, 2008; Paterson *et al*., 2009). Because of its use for fuel, feed, forage and food, sorghum has new market demands worldwide (Duff *et al*., 2019; Mundia *et al*., 2019). To provide for these needs, the function of its genes must be understood. Our large transcriptomic data sets for sorghum, grown under drought conditions in the field (Varoquaux *et al*., 2019; Xu *et al*., 2018), revealed that, although 44% of expressed genes were affected by drought, 43% of the transcriptome was not annotated (Varoquaux *et al*., 2019). This lack of foundational knowledge left questions about the function of genes involved in drought responses; however, lack of efficient transformation systems slows necessary experimentation to validate their function.

Engineering and editing are being used to study gene function, by overexpressing or turning off genes to modify traits in plants. However, using these methods for sorghum is challenging (Altpeter *et al*., 2016; Che *et al*., 2018). Since the first plant transformation success in tobacco (Bevan *et al*., 1983; Biotechnology and Biological Sciences Research Council, 2013; Fraley *et al*., 1983; Herrera‐Estrella *et al*., 1983), progress has been realized in crop and model plants. However, cereal crops have classically been more recalcitrant to Agrobacterium‐mediated transformation than dicots, largely due to the natural host of the pathogen being dicots (De Cleene and De Ley, 1976). Because of this, early maize success was accomplished using biolistics (Gordon‐Kamm *et al*., 1990).

Barriers to Agrobacterium‐mediated monocot transformation were eventually overcome in select species, rice being the first successful target (Raineri *et al*., 1990). However, Agrobacterium‐mediated transformation continues to be stymied in monocots by genotype dependence. Only specific cultivars within a species are amenable to transformation, limiting exploration of gene function across a species’ pangenome. Thus, elite agricultural varieties often cannot be transformed directly to modify traits. However, progress has been made in the once recalcitrant crop, maize (Frame *et al*., 2002; Shrawat and Lörz, 2006; Songstad *et al*., 1996). More recent efforts utilized specific morphogenes to significantly improve transformation efficiency (Lowe *et al*., 2016), a technique we call morphogene‐assisted transformation (MAT). Morphogenes used were *Zea mays Baby Boom* (*Zm‐Bbm, Opd2*), an AP2/ERF transcription factor that promotes cell proliferation during embryogenesis (Boutilier *et al*., 2002), and *Wuschel2* (*Wus2*), a transcription factor that maintains stem cells in the shoot meristem (Laux *et al*., 1996). Their overexpression, under strict regulatory control, generally sped transformation and overcame genotype dependence (Lowe *et al*., 2016), including recalcitrant maize varieties (Lowe *et al*., 2018; Mookkan *et al*., 2017) and several African sorghum varieties (Che *et al*., 2018).

Continued overexpression of morphogenes, however, causes negative phenotypic and reproductive outcomes, making excision or silencing of these genes necessary after inducing embryogenesis. In one approach morphogenes were embedded between *locus of crossing (x) over, P1* (*loxP*) sites and expression of CRE recombinase was triggered, causing excision of the morphogenes (Sauer and Henderson, 1988). Another approach in maize involved using two Agrobacterium strains, one with a gene‐of‐interest (GOI) construct and the other with a construct containing a morphogene. Using this strategy, termed altruistic, most transformants had the GOI but lacked morphogenes (Hoerster *et al*., 2020).

These successes led to the present study in sorghum, where *Zm‐Bbm* and *Wus2* were used in a MAT approach. This resulted in faster generation of transformants and more transformable U.S. sorghum genotypes: rapid‐cycling SC187 (Rosenow *et al*., 1997), stay‐green variety, BTx642, (Rosenow *et al*., 2002), BTx623 with the first sequenced genome (Frederiksen and Miller, 1972) (Paterson *et al*., 2009), and recalcitrant sweet sorghum Ramada (Freeman, 1974). To generate GOI‐containing plants, while simultaneously eliminating morphogenes to prevent aberrant phenotypes, necessitated a modified approach, termed altruistic MAT. In this approach an Agrobacterium with the morphogene construct was used to simultaneously infect with another Agrobacterium containing the GOI construct. Thus, altruistic MAT can be used to enhance efficiency of existing transformation constructs, as demonstrated in our study by plants transformed with only the RFP reporter plasmid, pANIC10A. Copy number determination was performed and, because of sorghum’s tendency to tiller, novel molecular genotyping methods were developed to assess transgene independent integration (TII). Finally, to facilitate gene function through targeted gene editing, morphogenes were included in a construct with a Clustered Regularly Interspaced Palindromic Repeats/CRISPR‐associated protein 9 (CRISPR/Cas9) cassette to edit an exemplary GOI, *phytoene desaturase* (*pds*). Together, advancements in this study provide a straightforward path to determine gene function in sorghum.

## Results

### Morphogene‐assisted transformation of multiple sorghum genotypes

**Figure 1:**
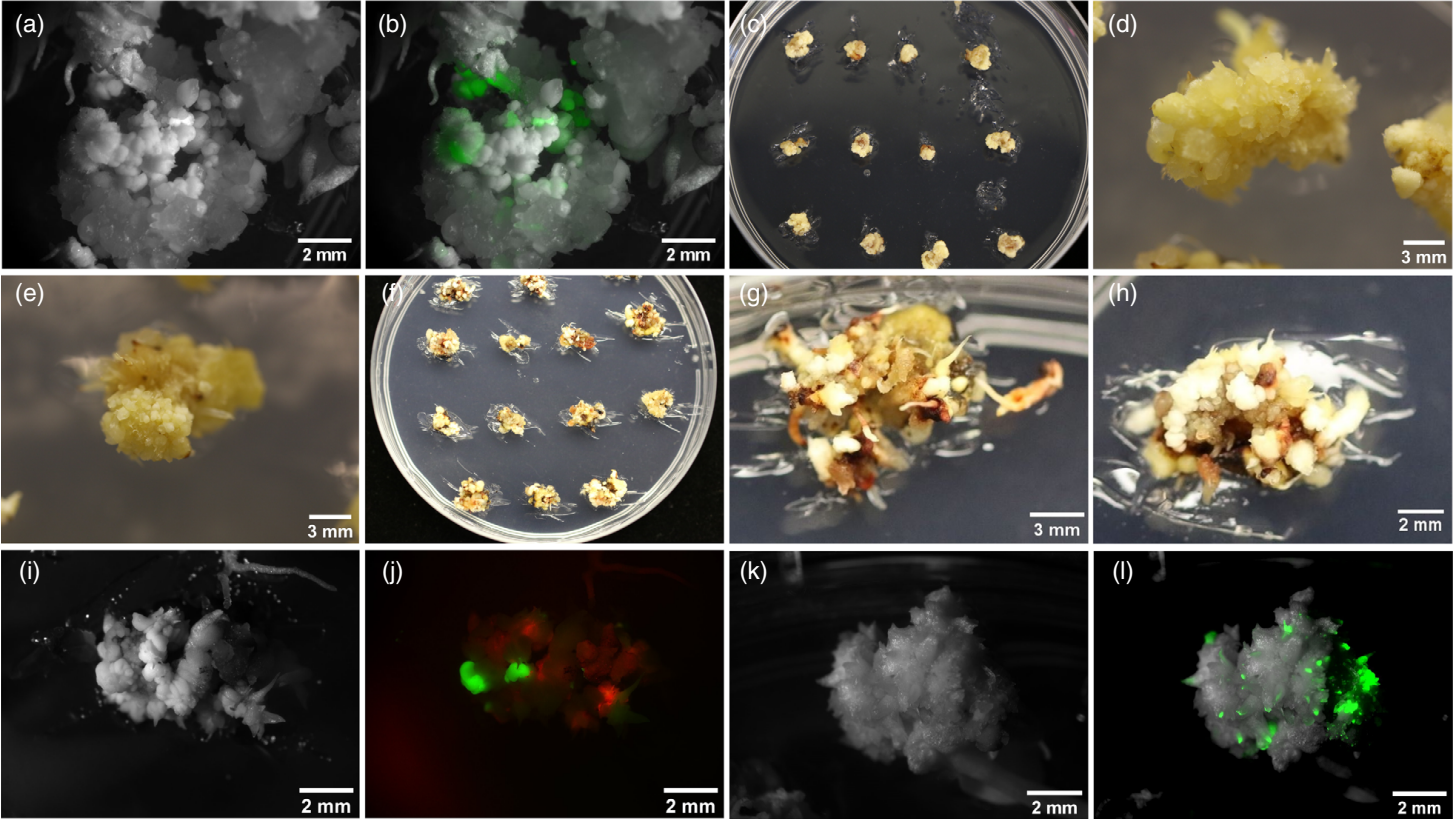
RTx430 tissues from morphogene‐assisted transformation (MAT) and altruistic MAT. RTx430 IEs, 1.5‐2mm, were transformed with Agrobacterium LBA4404 Thy‐ strain, containing appropriate constructs depending on the experiment. IEs were cultured for one week each on co‐cultivation then resting medium. For MAT, using pPHP81814 (ZSG), images by brightfield (a) and by fluorescence (b) were taken while tissues were on EMM. For MAT RTx430 tissues were moved to EMM with 0.05 mg/L imazapyr (IMZ) (c, d, e). For altruistic MAT 20 mg/L hygromycin was used as a selection agent for RTx430 (f, g, h). Images were taken using brightfield (i, k) and fluorescence microscopy (j, l). For altruistic MAT, using pGL190 (ZSG) and pANIC10A (RFP), IEs expressed ZSG and RFP (j). For comparison a brightfield image of the same tissue is shown (i). As a control, pGL190 was used to transform RTx430 IEs and imaged using brightfield (k) and fluorescence (l); ZSG expression in transformed tissue is shown (l).

To increase transformation speed and efficiency, we first used a MAT approach, using a single construct, pPHP81814 (Chu *et al*., 2019), containing the acetolactate synthase selection gene (*ALS*), the GOI for ZsGreen‐1 (*ZsG‐1*), and the morphogenes, *Zm‐Bbm* and *Wus2*. This resulted in transformation of five genotypes, RTx430, SC187, BTx642, BTx623 and Ramada. ZsG‐1 expression was visualized using fluorescence of immature embryos (IEs) between three and ten days post‐transformation, while tissues were on embryo maturation medium (EMM) (Figures 1a,b). Many somatic embryos strongly expressed ZsG‐1 (Figure 1b).

Imazapyr (IMZ), a systemic herbicide targeting ALS was used for selection on EMM and rooting medium (RM). RTx430 and BTx642 responded differently to selection, especially on EMM. RTx430 tissues, not killed on EMM with IMZ, formed more somatic embryos (Figures 1d,e), continuously forming tissue pieces about 1 cm in diameter. In contrast, BTx642 tissues on the same medium formed smaller numbers and sizes of somatic embryos (Figures S1a,b). Additionally, most RTx430 tissue under IMZ selection had some healthy tissues that were not brown or necrotic (Figures 1c,d,e) compared to BTx642 that did not (Figures S1a,b). On RM with IMZ, most untransformed plantlets became necrotic and died. When using IMZ selection, more escapes were observed relative to selection on hygromycin (hyg), used for altruistic MAT (see Altruistic morphogene‐assisted transformation).

### Genotyping of morphogene‐assisted transformation plants and transformation efficiency

After transfer to RM, putative transformants were genotyped using polymerase chain reaction (PCR). While transformation was successful for all genotypes, transformation efficiency, the number of T_0_ plants positive only for *ALS* without morphogenes, varied among genotypes (Table 1). Highest efficiency was for the most commonly transformed variety, RTx430 (9.4%), followed by rapid‐cycling SC187 (2.0%) and stay‐green variety, BTx642 (0.9%), neither previously reported as transformable. Also successful was BTx623 (0.1%), the first genotype with a complete genome sequence. One of the most recalcitrant varieties, sweet sorghum Ramada, has a published transformation efficiency of 0.09% (Raghuwanshi and Birch, 2010). In the present study, a 1.2% efficiency was achieved, an approximate 10‐fold improvement. Also noteworthy is the reduced time to produce T_0_ plants. The typical 18–21 weeks for RTx430 using conventional transformation procedures (Gurel *et al*., 2009; Wu *et al*., 2014) was reduced to 10–12 weeks.

**Table 1:** Morphogene‐assisted transformation efficiency in T_0_ plants of five sorghum genotypes transformed using pPHP81814 and IMZ selection

Genotype	# replications	# IEs used	# Regnerants	Escapes (%)^†^	+ for only *ALS* ^‡^	+ for only *ZSG* ^‡^	+ for *ALS* & *ZSG* ^‡^	Transformation efficiency (%)^§^	TII ^¶^ plants + for only *ALS* ^‡^	Transformation efficiency based on TII (%)^††^
RTx430	2	297	70	47.1	28	0	9	9.4	26	8.8
SC187	4	591	64	51.6	12	0	19	2.0	9	1.5
BTx623	2	783	25	84.0	1	2	1	0.1	1	0.1
BTx642	2	420	53	88.7	4	1	1	0.9	4	0.9
Ramada	3	850	60	76.7	10	2	2	1.2	5	0.6

^†^ Escapes % = percentage of regenerated plants without the transgene (T‐DNA).

^‡^ + indicates plants PCR‐positive for *ALS* and/or *ZSG*.

^§^ Transformation efficiency (%) = (plants + for only *ALS* divided by # IEs used) ×100.

^¶^ TII indicates transgene independent integration.

^††^ Transformation efficiency based on TII (%) = (TII plants + for only *ALS* divided by # IEs used) ×100.

**Figure 2:**
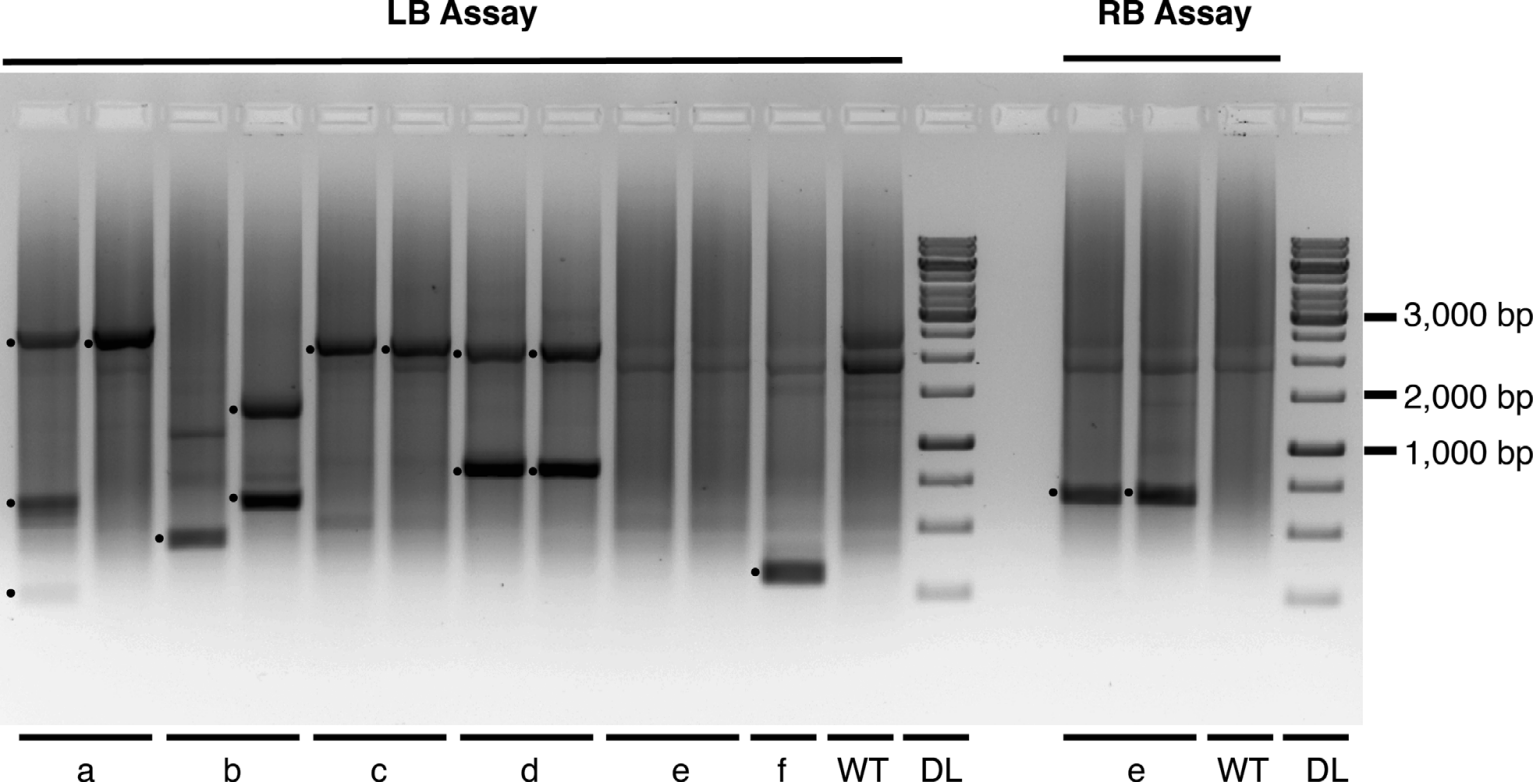
Transgene independent integration (TII) was determined with adaptor‐ligation‐mediated PCR. Examples of T‐DNA/gDNA junctions in the sorghum genome from the left border (LB, a‐f). Transgenic lines derived from the same immature embryo are labelled with alphabetic letters at the bottom; wild‐type genomic DNA (WT) is the negative control. Right border (RB) assay was performed if left border assay failed to amplify bands (e). DNA ladder (DL) is on the right. Dots indicate bands from T‐DNA/gDNA junctions. Plants from the same immature embryo in a and b are independent; plants in c, d and e are the same event.

While conventional sorghum transformation typically generates a single independent event per IE, use of *Zm‐Bbm* and *Wus2* led to numerous transgenic somatic embryos per IE, leading to T_0_ plants with unique transgene insertions. To confirm this, a method was devised to calculate efficiency, based on transgene independent integration (TII). This assured that multiple ALS‐positive plants with identical integration sites were not included when calculating efficiency. An inexpensive, high‐throughput method was used to determine the plants with TII. This was based on an adaptor ligation‐mediated PCR method, previously used for high‐throughput mapping in Arabidopsis (O’Malley *et al*., 2007). To modify this method, genomic DNA (gDNA) amounts were increased from 30 to 900 ng to account for sorghum’s larger genome size. To test this method, eleven transformed plants were chosen from six different IEs; one wild‐type plant was used as a negative control (Figure 2). Every sample, including the wild‐type (Lane 12), displayed two identical bands, possibly due to the number of amplification cycles, reinforcing the need to use genomic DNA without a T‐DNA insert as a negative control. The difference between the two methods of calculating transformation efficiencies for each genotype was small (Table 1), perhaps indicating efficiency of this transformation strategy.

When studying gene function, transgene copy number is also important for stable transgene expression. Digital droplet PCR (ddPCR) was used to quantify the number of inserted gene copies. Values were close to integer values (Table 2) and most were single‐copy: 63.2% of RTx430, 75.0% of SC187, and 77.8% of Ramada plants. After determining amplicon numbers for MAT transformants were mostly single‐copy, that analysis was found to be consistent with copy number analysis. This method generates multiple plants with independent insertions from numerous genotypes, critical for studying gene function and for complementing mutations.

**Table 2:** Transgene copy number in T_0_ plants of various sorghum genotypes using digital droplet PCR

Genotype	Construct	# of TII plants tested^†^	# single‐copy plants^‡^	# multi‐copy plants ^§^	% single‐copy plants^¶^
RTx430	pPHP81814	14	10	4	71.4
SC187		11	9	2	81.8
Ramada		9	7	2	77.8
RTx430	pGL190 + pANIC10A	40	25	15	62.5

^†^ # of TII plants tested = number of tested plants with transgene independent integration (TII).

^‡^ # single‐copy plants = number of plants with a single copy of *ALS* gene.

^§^ # multi‐copy plants = number of plants with multiple copies of *ALS* gene.

^¶^ % single‐copy plants = (# single‐copy plants divided by # T_0_ TII plants tested) × 100.

### Altruistic morphogene‐assisted transformation

Abnormal phenotypes due to introduced morphogenes (see Phenotypic characterization of putative transformed plants) prompted development of a new transformation strategy, modified from a maize method termed altruistic transformation (Hoerster *et al*., 2020). In this approach IEs were transformed simultaneously with two Agrobacterium strains, containing either pANIC10A (Mann *et al*., 2012), with the red fluorescent protein (RFP) GOI and the hygromycin phosphotransferase (*hph*) selection gene, or pPHP81814, in which WUS expression is driven by the relatively weak auxin‐inducible AXIG1 promoter (Jones *et al*., 2018).

**Figure 3:**
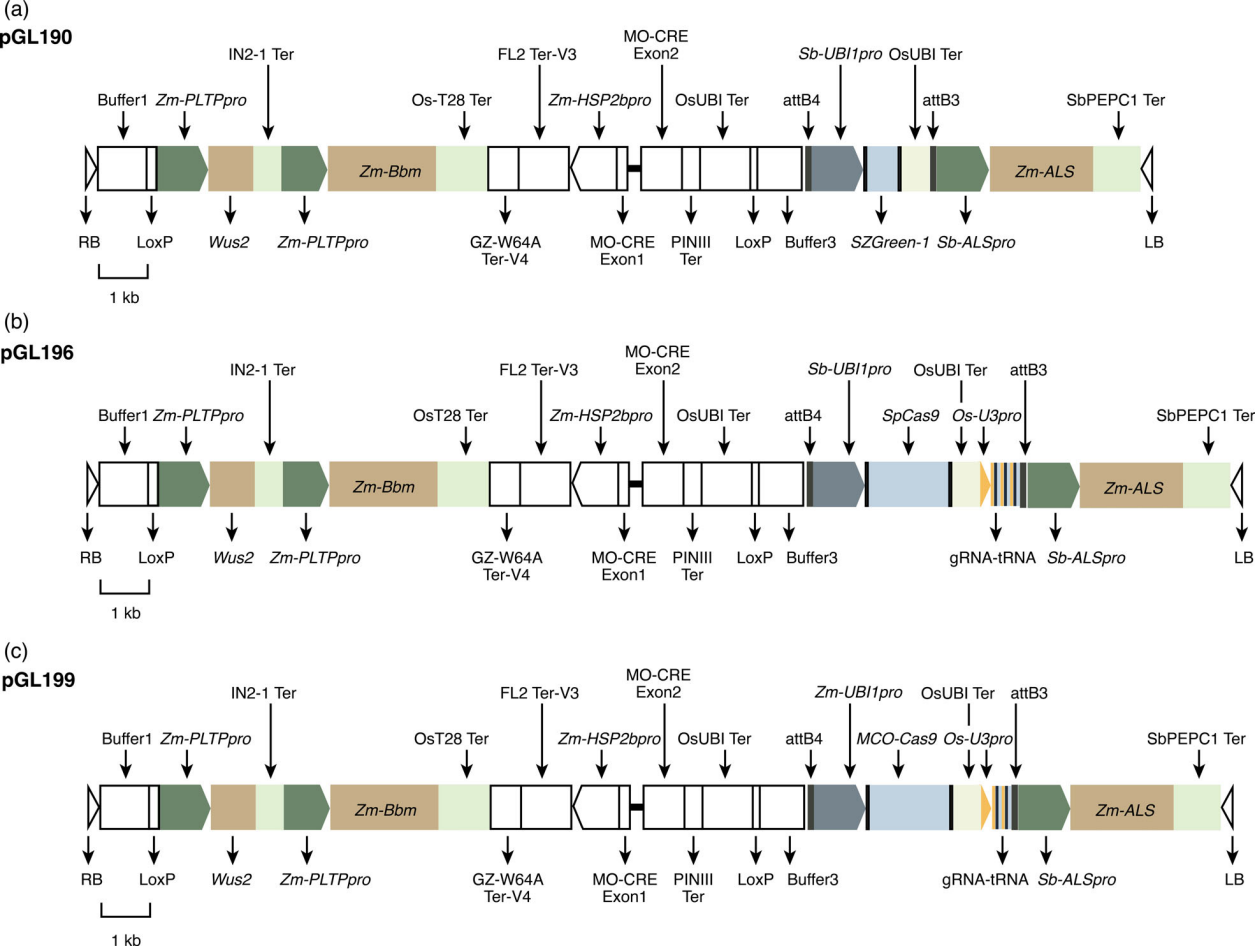
Construct maps for transformation. (a) pGL190 for altruistic morphogene‐assisted transformation (MAT). (b) pGL196 for MAT‐mediated editing of phytoene desaturase genes. The SpCas9 is driven by *Sb‐Ubi1pro*. Black rectangles indicate terminator regions. (c) pGL199 for MAT‐mediated editing of phytoene desaturase genes. The maize codon‐optimized SpCas9 is driven by *Zm‐Ubi1pro*. Triangles indicate T‐DNA borders; rectangles with arrows indicate promoters; blank rectangles indicate genes or buffer regions; Black rectangles indicate terminator regions. All three constructs were created from pPHP85425, as a Gateway‐compatible destination vector.

The first altruistic MAT experiment in RTx430 did not yield plantlets with the GOI. To improve this outcome in a second experiment another construct containing morphogenes was used. The first Agrobacterium strain contained pGL190 (Figure 3a), which has Zm‐Bbm and Wus2 and the ZsG‐1 marker gene. Expression of WUS is driven by a stronger maize promoter, PLTP (phospholipid transferase protein), compared to the promoter in pPHP81814 (Jones *et al*., 2018). The second strain had pANIC10A, with the RFP GOI and *hph*. The two strains were mixed 1:9 v:v, pGL190:pANIC10A, and applied simultaneously to sorghum IEs. Tissues were cultured on EMM plus hyg (Figures 1f–h) to select for hph expression; no selection was used on RM. Expression of ZsG‐1 from pGL190 and RFP from pANIC10A was visualized using brightfield (Figure 1i) and fluorescence microscopy (Figure 1j). Most IEs showed both ZsG and RFP expression 3 d post‐transformation. Transformations with only pGL190 (Figures 1k,l) or pANIC10A were used as controls. Transformants with the GOI were regenerated. Unlike IMZ selection, hygromycin selection in the altruistic MAT approach had more visible effects; growth of non‐transformed tissues was strongly inhibited, leadi ng eventually to necrosis. Effects of strong selection were also reflected in genotyping results (Table 3) where only 21.6% escapes were observed despite no selection on RM. The main difference between pGL190 and pPHP81814 constructs is the promoter driving Wus2. Since identical selection schemes were used, lack of success generating plants containing the GOI, when using pPHP81814 compared with pGL190, was likely due to the weaker promoter, Zm‐Axig1pro, driving Wus2 in pPHP81814.

**Table 3:** Efficiency of altruistic morphogene‐assisted transformation using pGL190 and pANIC10A in T_0_ plants

Genotype	# replications	# IEs used	# Regenerants	Escapes (%)^†^	+ for only *RFP & hph* ^‡^	+ for only *Bbm* ^‡^	+ for RFP, *hph* & *Bbm* ^‡^	Transformation efficiency (%)^§^	TII^¶^ plants + for only *RFP & hph* ^‡^	Transformation efficiency based on TII (%) ^††^
RTx430	4	379	232	21.6	113	18	51	29.8	47	12.4
SC187	1	83	20	0	10	0	10	12.0	9	10.8

^†^ Escapes (%) = percentage of regenerated plants without transgene (T‐DNA).

^‡^ + indicates plants PCR‐positive for *RFP* & *hph* (pANIC10A) and/or *Bbm* (pGL190).

^§^ Transformation efficiency (%) = (+ for only *RFP & hph* divided by # IEs used) × 100.

^¶^ TII indicates transgene independent integration.

^††^ Transformation efficiency based on TII (%) = (TII plants + for only *RFP & hph* divided by # IEs used) × 100

### Genotyping of altruistic morphogene‐assisted transformation plants and transformation efficiency

Similar to MAT, two different methods were used to calculate transformation efficiency, one reflecting the number of transformed plants and one reflecting the number of TII events. This permitted determination of whether transformants from the same IE were truly independent or were clonal events via tillering. For RTx430, four altruistic MAT replications were conducted using a total of 379 IEs (Table 3); for SC187, one replicate of 83 IEs was performed. PCR was used to detect *RFP, hph* and/or *Zm‐Bbm*. Transformation efficiency, the number of RTx430 T_0_ plants, positive only for *RFP and hph, was* 29.8% (113 of 379) and 12.0% (10 of 83) for SC187. Efficiency, based on TII, was 12.4% (47 of 379) for RTx430 and 10.8% (9 of 83) for SC187. For transgenic plants, 28.0% (51 of 182) of RTx430 and 50.0% (10 of 20) of SC187 plants contained *RFP, hph* and *Zm‐Bbm* (Table 3). As with MAT, if amplicons from T‐DNA left borders were not seen, junctions from right borders were used (Figure 2, sample e).

### Phenotypic characterization of putative transformed plants

Morphogenes were included in constructs to be able to transform more genotypes and to accelerate the process. But to regenerate plants with normal phenotypes and seed set, those genes must be removed from transformed tissues. For MAT with pPHP81814, putative transformed plants were tested using appropriate PCR primers (Table S1) to confirm CRE‐mediated excision of *ZsG‐1*, *Zm‐Bbm* and *Wus2*, accomplished using *loxP* sites flanking those genes, generating T_0_ plants positive only for *ALS* (Table 1). Genotypes showed different percentages of morphogene excision, with the highest rates for RTx430 (75.7%), Ramada (71.4%) and BTx642 (66.7%). Rates for BTx623 and SC187 were 25.0% and 38.7%, respectively. Plants negative for introduced morphogenes had normal growth and seed set; plants testing positive showed abnormal phenotypes, like short stature, twisted leaves and/or poor seed set (Figures S2a,b). For altruistic MAT with RTx430 and SC187, 62.1% and 50.0% of plants, respectively, had no morphogene insertion (Table 3) and normal phenotypes. Abnormal phenotypes were observed in plants with morphogenes.

### Genome editing mediated by MAT

In previous reports of sorghum editing, Agrobacterium‐mediated CRISPR/Cas9 delivery resulted in low efficiencies (Char *et al*., 2020; Ping Che *et al*., 2018; Li *et al*., 2018; G. Liu *et al*., 2019). Given the highly efficient sorghum transformation methods described in our study, use of MAT methods was combined with CRISPR/Cas9 editing to improve genome‐editing efficiency. Both MAT and altruistic MAT methods were considered to introduce the CRISPR/Cas9 cassette. The attempt chosen focused on MAT due to ease of construct manipulation and ability for T‐DNA segregation.

**Figure 4:**
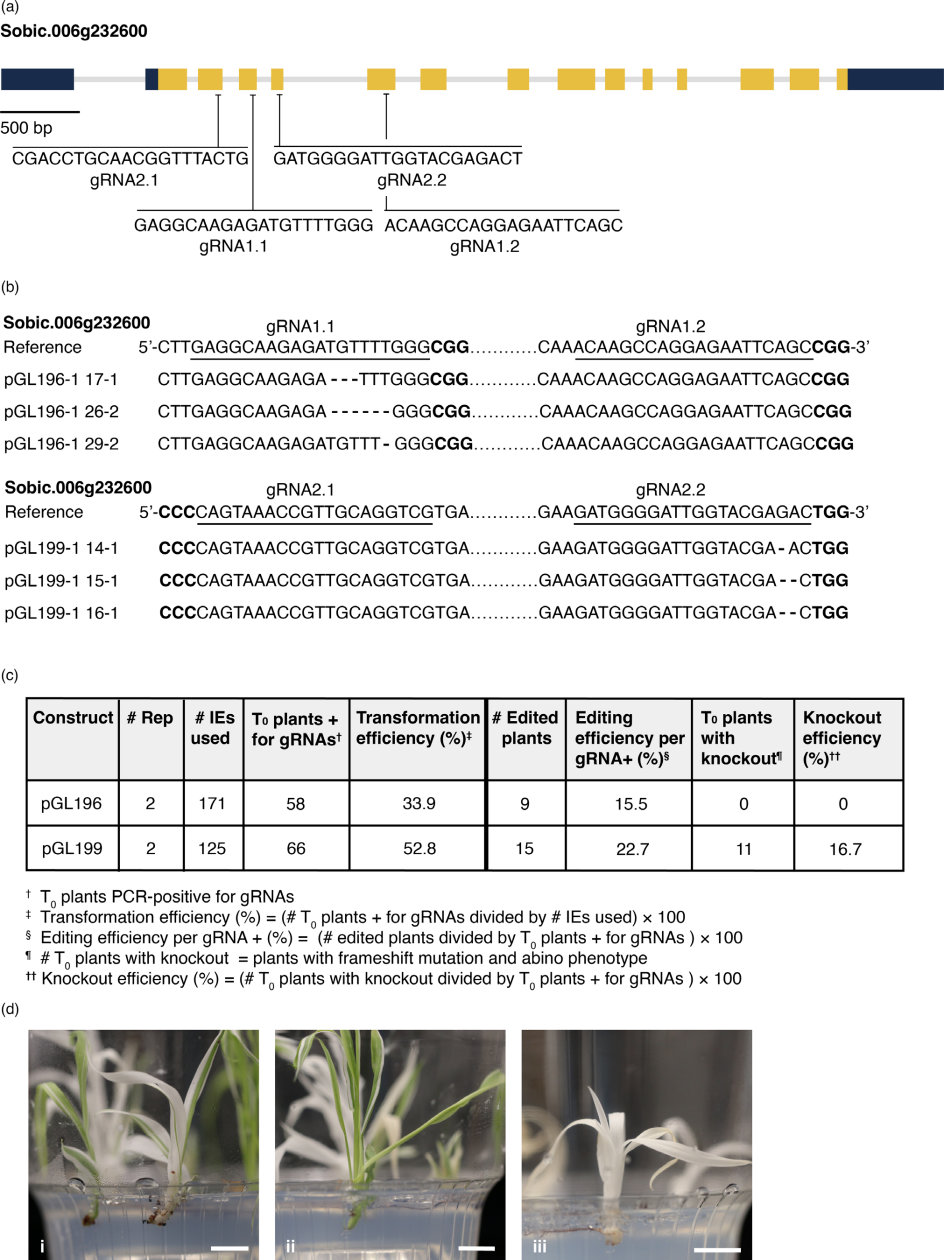
Editing of *pds* genes using CRISPR/Cas9 system. (a) Four guide RNAs (gRNAs) targeting *pds*. Dark blue rectangles indicate untranslated regions; yellow rectangles indicate exons; light grey lines indicate intron regions. gRNA1.1 is located in the third exon of *pds*. gRNA1.2 is located in the fifth exon of *pds*. These two gRNA are in pGL196. gRNA2.1 is located in the second exon of *pds*. gRNA2.2 is located in the fourth exon of pds. These two gRNAs are in pGL199. (b) Sanger sequencing of edited pds genes are shown with the main editing versions predicted by Synthego. gRNAs are underlined and PAM sites for gRNAs are in bold black font. Deletions are shown with dashes. (c) Summary of transformation efficiency and CRISPR/Cas9‐induced genome‐editing efficiency in T_0_ generation from MAT‐mediated editing in RTx430 using pGL196 and pGL199. (d) Albino phenotype from *pds* knockout, as frameshift mutations, in CRISPR/Cas9 edited plants. Phenotypic characterization of chimeric (i, ii) and fully albino (iii) mutants in T_0_ generation. Scale bar, 1 cm.

To create the CRISPR/Cas9 cassette, pGL196, the sorghum ubiquitin1 promoter (*Sb‐Ubi1pro*) was used to drive *Streptococcus pyogenes Cas9 (SpCas9)*. *Oryza sativa* U3 promoter (*Os‐U3pro*) was used to drive three gRNAs, targeting the two sorghum *pds* genes (Figures 4a, S4). The pGL196 construct also contained *Zm‐Bbm*, *Wus2* and the *ALS* selection gene (Figure 3b). pGL196 was introduced into RTx430. Transformed tissue was heat‐treated to trigger expression of heat‐shock‐inducible CRE, causing excision of morphogenes. Two replications using 171 IEs yielded 58 plants positive for gRNAs, a 33.9% transformation efficiency (Figure 4c). Of regenerants, no chimeric or biallelic albino phenotypes from a *pds* knockout were observed; however, genotyping revealed 9 of the 58 gRNA+ plants were edited, a 15.5% editing efficiency (Figure 4b).

To improve editing efficiency, Cas9 expression was increased as this likely impacted editing efficiency (Liu *et al*., 2019) and the maize, rather than the sorghum, ubiquitin1 promoter (*Zm‐Ubi1pro*) was used to drive maize codon‐optimized Cas9. This cassette was coupled in pGL199 with a cassette containing two *pds* gRNAs (Figures 3c, 4a). In two replications pGL199 was introduced into 125 IEs from RTx430 and 124 putative transformants were generated. PCR indicated that 66 plants were gRNA positive, giving a transformation efficiency of 52.8% (66/125) (Figure 4c). From gRNA‐positive plants, a *pds* fragment was amplified and analysed using Sanger sequencing and the Synthego Inference of CRISPR Edits (ICE) tool. Successful editing was detected in 15 of the 66 gRNA+ plants, yielding an editing efficiency of 22.7% (Figure 4c). Thus changes in pGL199 versus pGL196 resulted in higher editing efficiencies (22.7% versus 15.5%), increasing the probability of generating loss‐of‐function mutations (Figures 4c,d). Eleven of 15 edited plants had frame‐shift mutations, a knockout efficiency of 16.7% of the gRNA+ transformants (11 of 66) (Figure 4c). Of the 11 plants, six had a chimeric albino phenotype and five plants had a complete albino phenotype (Figure 4d); however, four did not survive after three weeks on RM. One chimeric plant survived and set seed.

## Discussion

To accelerate gene function identification in crops, transformation improvements are needed (Altpeter *et al*., 2016). This became obvious from our transcriptome data of drought‐stressed, field‐grown sorghum that revealed 44% of expressed genes were affected by drought, but only 57% of sorghum’s transcriptome is annotated (Varoquaux *et al*., 2019) and few annotated genes have validated functions. Major impediments exist in validating function in sorghum using classical methods of transformation, namely genotype dependence and the long times for selection, regeneration and generation advance. Efforts in this study aimed to address these issues.

### Morphogene‐assisted transformation reduces genotype dependence, improves efficiency and speed

In this study, morphogene‐containing constructs were used to demonstrate more sorghum varieties amenable to transformation. These include the first successful transformation of the prototype stay‐green variety, BTx642, allowing gene function studies on this important trait. Sweet sorghum Ramada transformation is reported at a rate approximately ten‐times that previously published (Raghuwanshi and Birch, 2010), enabling gene function studies in stem sugar production. Also notable was success with the fast‐cycling variety, SC187, for which T_1_ seeds are generated in the greenhouse ten weeks faster than with the standard transformation variety, RTx430. Newly published high‐quality BTx642 and SC187 Pac‐Bio sequences (Phytozome v^13^; Goodstein *et al*., 2012) will further increase success in gene function studies.

Classical sorghum transformation, requiring extended callus culturing, takes 15–18 weeks to generate RTx430 plantlets (Gurel *et al*. 2009; Wu *et al*., 2014). Implementing MAT strategies reported here accelerates regeneration of RTx430; T_0_ plants mature eight to nine weeks faster. This reduced time can be attributed to morphogenes directly inducing somatic embryogenesis in transformed tissue, as shown in maize where directed morphogene expression led to somatic embryos forming directly from surface cells of IEs (Lowe *et al*., 2018). This eliminates extended callus phases, which lengthen regeneration, leading to increased somaclonal variation (Bregitzer *et al*., 1998; Lemaux *et al*., 1999).

### Optimizing altruistic morphogene‐assisted transformation improves efficiency of existing transformation constructs

One challenge of MAT was generating transformed plants with the GOI, but without morphogenes, causing developmental abnormalities and poor seed set (Figure S2). This was addressed in maize using altruistic transformation (Hoerster *et al*., 2020). In this strategy high Wus2 expression in cells proximal to those with only the GOI yielded GOI‐positive transgenic plants without morphogenes. The *Zea mays* phospholipid transferase protein promoter (Zm‐PLTPpro) (Garnaat *et al*., 2002) drove strong Wus2 expression in scutellar epithelial cells of IEs (Lowe *et al*., 2018), such that sufficient Wus2 diffused into neighbouring cells, triggering developmental progression and GOI‐only transformed plants.

When pPHP81814 was used in our altruistic MAT method, the promoter driving Wus2 expression, *Zm‐AXIG1pro*, was weak and did not provide sufficiently strong expression to trigger developmental progression in neighbouring cells. Out of 192 IEs used, no plantlets containing the GOI were regenerated. Successful sorghum altruistic MAT necessitated using Zm‐PLTPpro to drive Bbm and Wus2 expression. In our efforts, presence in pANIC10A of the RFP GOI and *hph* required optimizing hyg selection. For altruistic MAT using pGL190 and pANIC10A, transformed RFP plants without morphogenes were generated; however, efficiencies varied among replicates. Many plants were also generated having both morphogenes from pGL190 and *hyg* and GOI from pANIC10A (Table 3). In maize, viral enhancers were used to increase Wus2 expression to levels likely hindering regeneration of plantlets with the morphogene (Hoerster *et al*., 2020). In our study, Wus2 expression likely was not sufficiently high to be toxic, leading to plants with morphogenes both with or without RFP and *hph*. Using this strategy, however, can increase transformation speed because previously completed GOI constructs can be used without having to insert morphogenes into those constructs. This allows researchers to use existing constructs to immediately improve transformation efficiency, expand transformable genotypes and expedite regeneration.

### Modified molecular genotyping techniques provide faster and more informative results

For transformed plants to provide definitive proof of gene function, copy number and TII must also be determined. ddPCR was used to determine copy number and, using MAT technology, notably over 60% of independent transformants were single‐copy. Determining TII in sorghum is also critical with engineering due to sorghum’s tendency to tiller, making it unclear if plants from the same IE are clonal or independent. Independence of events is less critical with CRISPR/Cas9 editing, however, because multiple plants from the same IE can have independent edits whether the original insertion is the same or not. Because previous methods to determine TII, like Southern hybridization and Southern‐by‐Sequencing (Zastrow‐Hayes *et al*., 2015), can be time‐ and resource‐intensive, a high‐throughput TII assay was created by modifying a transgene insertion mapping method developed for Arabidopsis (O’Malley *et al*., 2007). Using PCR‐based strategies to determine both copy number and TII, the large number of transformed plants generated by MAT can be genotyped at a higher throughput rate with reduced material and personnel costs.

### Integrating morphogene‐assisted transformation and CRISPR/Cas9 gene editing produces biallelic and chimeric T_0_ knockouts

Previous reports in sorghum described success with editing endogenous genes using both biolistic bombardment and Agrobacterium‐mediated transformation (Char *et al*., 2020; Che *et al*., 2018; Li *et al*., 2018; G. Liu *et al*., 2019). To improve both transformation and editing efficiencies in sorghum, MAT was combined with CRISPR/Cas9, using multiple gRNAs to edit the exemplary GOI, *pds*. Based on pGL196 editing experiments (Figure 4c), the altruistic MAT strategy yielded tissues with fewer somatic embryos that proliferated more slowly than when using MAT alone. This led to the decision not to use an altruistic MAT approach for editing, opting instead to combine morphogene cassettes with a CRISPR/Cas9 cassette.

Using pGL196, numerous transformants were identified in which morphogenes were not integrated; however, albino phenotypes due to biallelic *pds* knockouts were not observed. Low editing efficiency with pGL196 (Figure 4c) was likely due to low‐level expression of Cas9 driven by *Sb‐Ubi1pro*, compared to the maize ubiquitin1 promoter (*Zm‐Ubi1pro*) (I. Godwin, personal communication). Replacing *Sb‐Ubi1pro* and *SpCas9* with *Zm‐Ubi1pro* and maize codon‐optimized *SpCas9*, respectively, in pGL199 (Figure 3c) likely contributed to increased editing efficiency due to higher expression levels, leading to regeneration of albino plantlets, due to biallelic editing in the T_0_ generation (Figure 4d). This is the first report of biallelic edits in T_0_ sorghum plants using CRISPR/ Cas9.

Introducing a single T‐DNA, including gRNAs, *Cas9*, LoxP and morphogenes (pGL199) led to 16.7% knockout efficiency (Figure 4c); however, there are strategies to increase rates of heritable editing mutations, for example, using a meiosis‐specific promoter, like dmc1, to drive Cas9, as demonstrated in maize (Feng *et al*., 2018). Another approach might be to use Cas9 orthologues or variants, like *Streptococcus thermophilus Cas9* (*StCas9*) (Müller *et al*., 2016) and *Staphylococcus aureus* Cas9 (*SaCas9*) (Ran *et al*., 2015). Despite our demonstrated success, integrating MAT and CRISPR/Cas9 into a single construct is not without disadvantages. The resulting plasmid is very large, ~36 kb, and creation of plasmids this size and their introduction into Agrobacterium can be difficult (Table S4). Also having the GOI and morphogenes on the same T‐DNA necessitated removing introduced morphogenes using heat shock to generate plants with normal phenotypes and seed sets (Table 1). Despite these challenges, the considerable improvements in transformation efficiency and the reduced regeneration time observed with the MAT approach, compared to classical transformation, demonstrate its efficacy in facilitating CRISPR/ Cas9 gene editing experiments in sorghum.

## Conclusion

Certain steps outlined in this study facilitated development of a pathway from creation and introduction of target genes via engineering and editing to a means to determine gene function. Use of morphogenes led to more sorghum genotypes amenable to transformation and a faster time to generate transformed plants. Another challenge facing researchers with limited resources is garnering the technological resources necessary to get from gene identification to transformed plants ready for field trials. A path to achieving this goal is described in the present study by bringing together existing and newly created constructs, culturing approaches, and analytical methods. Details are provided on these resources and methods – including transformation of new sorghum genotypes, identification of single‐copy, independent events, and creation of MAT‐generated editing constructs. These advancements provide a straightforward means to validate gene function in sorghum for the many uncharacterized genes (Phytozome v^13^), including those involved in drought responses (Varoquaux *et al*., 2019; Xu *et al*., 2018).

## Methods

### Plant materials

Seeds from sorghum varieties RTx430 (Miller, 1984), BTx623 (Frederiksen and Miller, 1972), BTx642 (Rosenow *et al*., 2002), SC187 (Rosenow *et al*., 1997) and sweet sorghum Ramada (Freeman, 1974) were from GRIN (United States Department of Agriculture, Agricultural Research Service, n.d.), planted in three‐gallon pots with SuperSoil 1 (Rod McClellan Co., South San Francisco, CA) and grown in the greenhouse at 28 °C with 16 h light/ 8 h dark photoperiod. IEs from panicles were collected at 12–14 days post‐anthesis.

### Preparation of Agrobacterium

LBA4404 Thy‐ is an auxotrophic (THY‐) *A. tumefaciens* strain (Anand *et al*., 2017) into which helper plasmid, pPHP71539 (Anand *et al*., 2017), was introduced. For other constructs, strains were streaked on YEP agar medium (per L: 10 g yeast extract, 10 g Bacto Peptone, 5 g NaCl, 15 g Bacto Agar, pH 7.0, 100 mg/L thymidine, 50 mg/L gentamicin) from stocks stored at −80 °C at 25% final concentration glycerol. After dark incubation for 3 days at 28 °C, 3–5 colonies were used to make overnight cultures on YEP; suspension cultures from those cultures were adjusted to OD_550_ 0.7, using PHI‐I medium (Wu *et al*., 2014) with 0.005% silwet and 0.2 mM acetosyringone.

### Transformation constructs

Primers for creating constructs are in Table S2. pPHP81814 (Chu *et al*., 2019), used for MAT, has Zm‐*Bbm* (Boutilier *et al*., 2002) *and Wus2* (Nardmann and Werr, 2006) plus screenable marker gene ZsGreen‐1 (*ZsG‐1*), driven by *Zm‐Axig1pro*, *Zm‐PLTPpro*, and *Sb‐Ubi1pro*, respectively. These three genes are within loxP sites; P4 recombinase gene causing recombination (*cre*) is driven by the maize late embryogenesis‐stage *Zm‐GLB1pro* (Belanger and Kriz, 1989). *ALS* is driven by the *Sorghum bicolor* acetolactate synthase promoter (*Sb‐ALSpro*). Helper plasmid, pPHP71539, contains *virB*, *virC*, *virD*, *virE* and *virG* and a bacterial gentamicin resistance gene (Anand *et al*., 2018).

To create altruistic MAT construct, pGL190 (Figure 3a), pPHP83911 (Figure S3a), contained *Sb‐Ubi1pro* and the first intron driving *uidA* and the sorghum gamma kafirin terminator, linearized between attL3 and attL4, using pPL01 and pPL04 primers. The expression cassette, including the *Sb‐Ubi1pro* and first intron, *ZsG‐1*, and the rice *Ubi1* terminator, was inserted using pPL02 and pPL03 primers via Gibson Assembly (New England BioLabs, Ipswich MA). Entry vector was verified by Sanger sequencing with primers pPL02 and pPL05, and M13 reverse primers. To complete pGL190 construction, the entry vector was recombined with the destination vector, pPHP85425 (Figure S3b), confirmed by *BamHI* restriction and electroporated into LBA4404 Thy‐. Plasmid was confirmed by complete plasmid sequencing (Center for Computational and Integrative Biology, Massachusetts General Hospital, Boston MA).

For pGL193 (Figure S3c), pPHP83911 (Figure S3a) was linearized with pPL08 and pPL09 primers. SpCas9 was amplified from pRGEB32 (Xie *et al*., 2015) with pPL06 and pPL07 primers and the two fragments ligated via Gibson Assembly. The gRNA expression cassette, amplified from pRGEB32 with pPL10 and pPL11 primers, was inserted via Gibson Assembly into *Sma*I‐linearized plasmid. The guide RNAs (gRNAs) polycistronic cassette, targeting two sorghum *pds* exons (Figures 4a, S4), created with primers pPL12 to pPL17 according to the published protocol (Xie *et al*., 2015). Using Golden Gate Assembly, the gRNA‐tRNA polycistronic cassette was digested with *Fok*I, and ligated into pGL193. pGL196 (Figures 3b, S5) was generated by recombining the *pds* gRNA‐tRNA polycistronic cassette and the SpCas9 gene from pGL193 with pPHP85425). For pGL198, maize *Ubi1* promoter and maize codon‐optimized *SpCas9* were amplified from pBUN411 (Xing *et al*., 2014) with primers pPL27 and pPL28, replacing by Gibson Assembly the *Sb‐Ubi1pro* and SpCas9 gene in pGL193 (Figure S3c). Two gRNAs, targeting *pds* genes in the gRNA‐tRNA polycistronic cassette, were assembled into pGL198 using primers pPL29 to pPL32, with the method described above. pGL199 (Figures 3c, S5) was generated by recombining the *pds* gRNA‐tRNA polycistronic cassette and maize codon‐optimized SpCas9, with destination vector, pPHP85425. All constructs were confirmed by Sanger sequencing. pGL190 (Figure 3a), pGL196 (Figure 3b), and pGL199 (Figure 3c) from Agrobacterium were confirmed by complete plasmid sequencing (Center for Computational and Integrative Biology, Massachusetts General Hospital, Boston MA).

### Morphogene‐assisted transformation of sorghum

All media was modified from (Jones *et al*., 2019) (Table S3). General construct descriptions and plant and bacterial selection genes and their purpose are in Table S4. Immature seeds of RTx430, BTx623, BTx642, SC187 and Ramada from greenhouse‐grown plants were surface‐sterilized twice for two mins with 75% ethanol, then 20% bleach plus 0.2% Tween20 for 20 mins. IEs (1.5–2 mm) were isolated and put into PHI‐I liquid medium. When all IEs were isolated, PHI‐I was removed, 1 mL of Agrobacterium suspension was added and mixed 5 mins at medium shaker speed; suspension was removed by pipetting. IEs, placed on co‐cultivation medium (CCM) scutellum side up, were kept in the dark for 7 days at 24 °C. IEs were transferred to resting medium for 7 days to halt Agrobacterium growth; tissues were then moved to embryo maturation media (EMM) with 0.05 mg/L IMZ (Sigma Aldrich Chemicals, St. Louis MO) until shoots formed (Figures S1a,b). Plantlets (2–3 cm) were moved to RM with 0.05 mg/L IMZ under 16‐h photoperiod at 26 °C (Figure S1c). Plants with well‐established roots and shoots (10‐12 cm) were moved to soil in growth chambers at 26 °C with 16‐h light and 8‐h dark photoperiod for two weeks of conditioning before transferring to the greenhouse. Per cent transformation efficiency was calculated in two ways. First, efficiency was based on the number of T_0_ plants PCR‐positive for the target gene, divided by the number of IEs used, times 100. The second method was based on the number of PCR‐positive, TII T_0_ plants.

### Altruistic morphogene‐assisted transformation of sorghum

Immature RTx430 seeds were harvested, sterilized and IEs excised as described for the MAT approach. For transformation, Agrobacterium LBA4404 Thy‐, containing either pGL190/pPHP81814 or pANIC10A at OD_550_ 0.7, were mixed in a 9:1 (pANIC10A: pGL190/pPHP81814) volume: volume ratio. IEs were cultured as described for MAT except EMM selection was on 20 mg/L hygromycin (hyg; PhytoTechnology Laboratories, Lenexa KS); no selection in RM. Brightfield and fluorescence microscopy images were taken. pGL190 and pANIC10A alone were used as transformation controls.

### DNA extraction

Leaf samples, ~3 cm, frozen in liquid nitrogen and ground in an MM300 bead beater (Retsch GmbH, Haan Germany) for 1.5 mins at 25 cps, were refrozen and ground again. 700 μl of urea buffer (2 M urea, 0.35 mM NaCl, 20 mM Tris‐HCl pH 8, 20 mM EDTA, 1% sarkosyl) was added and vortexed for 30 secs. 10 μl of RNase A was added, incubated 10 mins at room temperature and 700 μl of phenol:chloroform:isoamyl alcohol (25:24:1) was added, vortexed 15 mins, and centrifuged 13 800 × *g* for 15 mins. 55 μl of 3 M sodium acetate (pH 5.2) and 367 μl of isopropanol were added to 550 μl of the aqueous phase; to precipitate DNA, tubes were inverted at −20 °C overnight or −80 °C for 1 h. After centrifugation at 13 800 × *g* for 5 mins, supernatant was removed and pellet rinsed in 500 μl 70% ETOH. Samples were centrifuged 5 mins at 13 800 × *g*, supernatant removed and samples centrifuged 1 min. Excess ethanol was removed, pellets air‐dried 5–10 mins in laminar flow hood, resuspended in sterile distilled water and stored at −20 °C.

### Fluorescence visualization

Zeiss Lumar v12 epifluorescence stereomicroscope and a QImaging Retiga SRV camera were used to observe IEs on EMM expressing *RFP* and *ZsG*‐1 between 3 and 10 days post‐infection and while tissues were on EMM. RFP and ZsG‐1 fluorescence was detected using Texas Red and Endow GFP filter sets, respectively.

### PCR analysis of putative transformed plants

Polymerase chain reaction was performed on gDNA from leaf tissue of putative T_0_ plants. For putative, MAT‐generated, PCR primers (Table S1) were used to confirm *ZsG‐1* and *ALS*, reinstated after CRE‐mediated excision of Zm‐*Bbm* and *Wus2*. For the altruistic MAT approach with pGL190 and pANIC10A, PCR was performed using *hph, RFP* and *Bbm* primers (Tables S1, S5).

### Transgene copy number analysis

For transgene copy number, primers and HEX™‐labelled probes were designed to detect the endogenous reference gene, protein phosphatase 2A (PP2A) (Table S6), chosen as one of the most stable in sorghum in different tissues and stress treatments (Sudhakar Reddy *et al*., 2016). This was used for RTx430, SC187 and Ramada. A different primer set (Table S6) was used to detect *ALS*, using probes labelled with FAM™ and double‐quenched with ZEN™ and Iowa Black Hole Quencher^®^ (Integrated DNA Technologies, Inc., Coralville, IA). Probes and primers were designed according to the manufacturer's protocol (BioRad Laboratories, Hercules CA); droplets were generated using QX200 droplet generator. Samples included a no template control (ddH20), negative control (wild‐type sorghum gDNA), positive plasmid DNA and gDNA from transformed plants. HindIII, which does not cut the transgene, was used to digest gDNA. Samples were transferred to 96‐well plates; a thermal cycler was used for PCR amplification. To optimize quantification of transgene copy number, gradient PCR (55–65 °C) and dilution gradient of sorghum DNA (6.8–55 ng) was used to determine optimal annealing temperatures and DNA amounts; 55 °C and 27.5 ng gDNA were selected because they gave the highest fluorescence amplitude difference between negative and positive droplets, specific amplification and clear separation between negative and positive droplets. PCR started with 95 °C for 10 mins, 94 °C for 30 secs and 55 °C for 1 min; each step was 40 cycles and 2 °C/second ramp rate. Reactions were stopped by 98 °C enzyme deactivation for 10 mins. Fluorescence was measured using BioRad QX200 Droplet reader; more than 10 000 droplets were generated per sample. Samples were performed in duplicate; data was analysed using BioRad QuantaSoft™ Software.

### TII determination

All primers in this section are in Table S2. The TII method was modified from (O’Malley *et al*., 2007). Adaptors for *Hin*dIII (adpH3) and *Bam*HI (adpB1) were annealed with pPL18 and pPL19, plus pPL18 and pPL20, respectively. Oligonucleotides were heated at 98 °C, then cooled to room temperature by turning the water bath off. 900 ng of sorghum gDNA was added to adpH3 and adpB1 adapters, using *Hin*dIII and *Bam*HI and T4 ligase; the reaction was at 25 °C for 16 h in a thermocycler (BioRad C1000 Touch™). To analyse gDNA‐tDNA junctions on the left border for MAT‐generated transformants, PCR amplification was performed using pPL21 and pPL23 primers at 55 °C for annealing; elongation was for 4 min. If distinct PCR bands were not seen, PCR products were used for nested‐PCR amplification. 1 μl of PCR product was amplified at 60 °C for annealing, elongation for 4 min using pPL24 and pPL26 primers. PCR products were analysed in 1% agarose and read using Image Lab Software (BIO‐RAD, Hercules CA). If the Transfer‐DNA (T‐DNA)/genomic DNA (T‐DNA/gDNA) junctions could not be amplified from left borders, ligates were amplified from right border with primers pPL22 and pPL23 for first‐round PCR, pPL25 and pPL26 for nested PCR, if needed. Left or right border data was used to determine TII (Figure S6). For MAT‐generated transformants, TII was determined as for the samples from altruistic MAT by amplifying T‐DNA/gDNA junctions using primers pPL23 and pPL33 for first round PCR, primers pPL26 and pPL34 for nested PCR. For TII, it is not mandatory to first verify plants are single‐copy; however, amplification and visualization of gel images are easier.

### CRISPR/Cas9 editing mediated by morphogene‐assisted transformation

To demonstrate editing efficiency, three gRNAs targeting *pds,* Sobic.006G232600 on chromosome 6, and a homologue, Sobic.001G480550 on chromosome 1 [88%, 90% identity, respectively, to rice *pds* LOC_Os03g08570 coding sequence (Phytozome v^13^)], were designed according to CRISPR‐P 2.0 (H. Liu *et al*., 2017) (Figures 4a, S4). The first gRNA targeted the third exon of both genes; the second and third gRNAs targeted the fifth exon of both *pds* genes. Three gRNAs were assembled into pGL193 (Figure S3c) for LR recombination (Figure S5). For gRNA optimization, two gRNA sequences targeting the second and fourth exons of *pds*, Sobic.006G232600 (Figure 4a), were from Dr. Ian Godwin (personal communication). gRNAs were incorporated in pGL198 (Figure S3d) for subcloning (Figure S5). For MAT‐mediated editing, SpCas9 expression in the expression cassette was driven by *Sb‐UBi1pro* or *Zm‐Ubi1pro*. respectively, in pGL193 or pGL198; gRNA‐tRNA polycistronic expression was driven by *Os‐snoRNAU3pro* on the pENTR vectors (Figures S3c,d). After incorporating gRNAs targeting *pds* (Figures 4a, S4) into both cassettes they were introduced into the destination vector, pPHP85425 (Figure S3b), yielding, pGL196 and pGL199 (Figures 3b,c, S5), which were transformed into RTx430 using *Agrobacterium tumefaciens* LBA4404 Thy‐. Before moving to EMM, IEs on resting media were heat‐shocked at 45°C, 75% humidity, for 2 hrs to trigger CRE‐mediated excision. From each IE, 3‐4 plantlets were moved to RM.

### Genotyping putative CRISPR/Cas9 edited plants

Total gDNA was extracted from leaves of seedlings on RM. To identify putative edited plants, PCR was performed using primers for the gRNA‐tRNA polycistronic cassette and Zm‐*Bbm* (Table S1). Knockout of Sobic.006G232600 leads to albino phenotypes (G. Liu *et al*., 2019), so the target *pds* fragment, including the two gRNAs, was amplified to analyse by Sanger sequencing. Targeted *pds* genes were amplified in PCR‐positive plants, using PDS‐06g‐1 primers, to detect putative edits with pGL196, PDS‐06g‐2 primers for putative edits with pGL199 (Table S1). Amplicons were visualized in 1% agarose, purified with QIAquick^®^ PCR purification kit (QIAgen, Redwood City CA), and prepared for Sanger sequencing (UC Berkeley DNA Sequencing Facility). Sequencing results were analysed using the Inference of CRISPR Edits (ICE) analysis tool (Hsiau *et al*., 2018).

## Conflict of interest statement

pPHP vectors are available for research purposes; using the vectors for commercial applications requires a paid non‐exclusive license from Corteva Agriscience. Manuscript authors have no relationship with Corteva Agriscience.

## Author contributions

Kiflom Aregawi: Investigation, Transformation Methodology, Writing ‐ Original Draft ‐ Review & Editing. Jianqiang Shen: Investigation, Transformation Methodology, Constructs, Writing ‐ Review & Editing. Grady Pierroz: Investigation, Transformation Methodology, Writing – Review & Editing. Manoj Sharma: Transformation Methodology, Writing ‐ Review & Editing. Jeffery Dahlberg: Methodology. Judith Owiti: Transformation Methodology, Writing ‐ Review & Editing. Peggy G. Lemaux: Conceptualization, Formal analysis, Supervision, Project administration and funding acquisition, Writing – Original Draft –Review & Editing.

## Supporting information


**Figure S1**. (a, b) BTx642 tissues on EMM with IMZ selection following transformation with pPHP81814. (c) Plantlets growing on rooting media with IMZ.
**Figure S2**. Plants from (a) RTx430 and (b) BTx642 transformed with pPHP81814. In both, plants null for *Zm‐Bbm* and *Wus2* (right) and plants retaining introduced *Zm‐Bbm* and *Wus2* (left) have shorter stature, twisted leaves and poorer seed set, compared to wild‐type.
**Figure S3**. Schematic representation of the molecular components of constructs. (a) pPHP83911, used for generating pGL193 and pGL198, is a pENTR vector. (b) pPHP85425 is a destination vector for morphogene‐assisted transformation (MAT). Molecular components between left and right borders are shown. (c) pGL193 is a pENTR vector for MAT‐mediated editing with gRNAs. The remaining backbone part is from pPHP83911. (d) pGL198 is a pENTR vector for MAT‐mediated editing with the maize ubiquitin promoter and maize codon‐optimized SpCas9 for carrying gRNAs. The remaining backbone part is also from pPHP83911.
**Figure S4**. Two gRNAs targeting *pds* in pGL196; yellow rectangles indicate exons; light grey lines indicate intron regions. gRNA1.1 is located in the third exon of *pds* genes. gRNA1.3 is located in the fifth exon of *pds* genes.
**Figure S5**. Molecular cloning strategy for constructing sorghum CRISPR/Cas9 vectors. Cloning vector for gRNAs containing *Bsa*I sites for sequential insertion of two or three gRNAs with tRNAs that forms polycistronic cassettes (gRNA‐tRNA), driven by rice U3 promoter (*Os‐U3pro*). The gRNA‐tRNA and Cas9 expression cassettes were mobilized into pPHP85425 through Gateway recombination, generating pGL196 and pGL199 for Agrobacterium‐mediated sorghum transformation.
**Figure S6**. Determination of transgene independent integration (TII) with adapter ligation‐mediated PCR. Genomic DNA with a T‐DNA insert (i) was digested with *Eco*RI and *Hin*dIII (ii). Adapters are ligated to digested sites creating adapter‐flanked templates (iii). Only the longer arm of the adapter contains a sequence exactly matching the adapter primers. Adapter‐to‐adapter amplification does not occur due to lack of primer match sites on the shorter arm of adapters. If T‐DNA is present in the template (black line, Figure 4a.i), T‐DNA primers (yellow arrow, left border; dark blue arrow, right border, Figure 4a.iii) will bind to their corresponding sites and initiate synthesis of a complementary strand (iv). PCR products will contain adapter primer‐binding sites derived from the complement of the longer arm of the adapter.
**Table S1**. Primers for genotyping putative transformed plants
**Table S2**. Primers for constructs, gRNAs and determining transgene independent integration (TII)
**Table S3**. Medium composition for sorghum transformation
**Table S4**. Constructs used for sorghum transformation
**Table S5**. PCR programmes for genotyping putative transformed plants
**Table S6**. Primers and probes for digital droplet PCR
**Table S7**. Description and references for construct elements used for transformation

## Data Availability

As available, accession numbers and gene names are provided in text and Table S7. Methods for generating different constructs and transformation protocols are described in the text, Supplementary Tables and Supplementary Figures. PHP vectors are freely available for research; commercial applications require a paid non‐exclusive license from Corteva Agriscience. The sequences of pGL193 and pGL198 were deposited to GeneBank, with the accession numbers, OK017460 and OK017461 respectively. Both of them will be deposited and available on Addgene as well.
