# Supplementary material for: Morphogene‐assisted transformation of Sorghum bicolor allows more efficient genome editing
Source: Plant Biotechnol J. 2021 Dec 16;20(4):748–60. doi: 10.1111/pbi.13754 (PMC8989502; doi:10.1111/pbi.13754)
Supplement: Supplementary file 1 — Figure S1. (a, b) BTx642 tissues on EMM with IMZ selection following transformation with pPHP81814. (c) Plantlets growing on rooting media with IMZ. Figure S2. Plants from (a) RTx430 and (b) BTx642 transformed with pPHP81814. In both, plants null for Zm‐Bbm and Wus2 (right) and plants retaining introduced Zm‐Bbm and Wus2 (left) have shorter stature, twisted leaves and poorer seed set, compared to wild‐type. Figure S3. Schematic representation of the molecular components of constructs. (a) pPHP83911, used for generating pGL193 and pGL198, is a pENTR vector. (b) pPHP85425 is a destination vector for morphogene‐assisted transformation (MAT). Molecular components between left and right borders are shown. (c) pGL193 is a pENTR vector for MAT‐mediated editing with gRNAs. The remaining backbone part is from pPHP83911. (d) pGL198 is a pENTR vector for MAT‐mediated editing with the maize ubiquitin promoter and maize codon‐optimized SpCas9 for carrying gRNAs. The remaining backbone part is also from pPHP83911. Figure S4. Two gRNAs targeting pds in pGL196; yellow rectangles indicate exons; light grey lines indicate intron regions. gRNA1.1 is located in the third exon of pds genes. gRNA1.3 is located in the fifth exon of pds genes. Figure S5. Molecular cloning strategy for constructing sorghum CRISPR/Cas9 vectors. Cloning vector for gRNAs containing BsaI sites for sequential insertion of two or three gRNAs with tRNAs that forms polycistronic cassettes (gRNA‐tRNA), driven by rice U3 promoter (Os‐U3pro). The gRNA‐tRNA and Cas9 expression cassettes were mobilized into pPHP85425 through Gateway recombination, generating pGL196 and pGL199 for Agrobacterium‐mediated sorghum transformation. Figure S6. Determination of transgene independent integration (TII) with adapter ligation‐mediated PCR. Genomic DNA with a T‐DNA insert (i) was digested with EcoRI and HindIII (ii). Adapters are ligated to digested sites creating adapter‐flanked templates (iii). Only the longer arm of the adapter c [file PBI-20-748-s001.pdf]

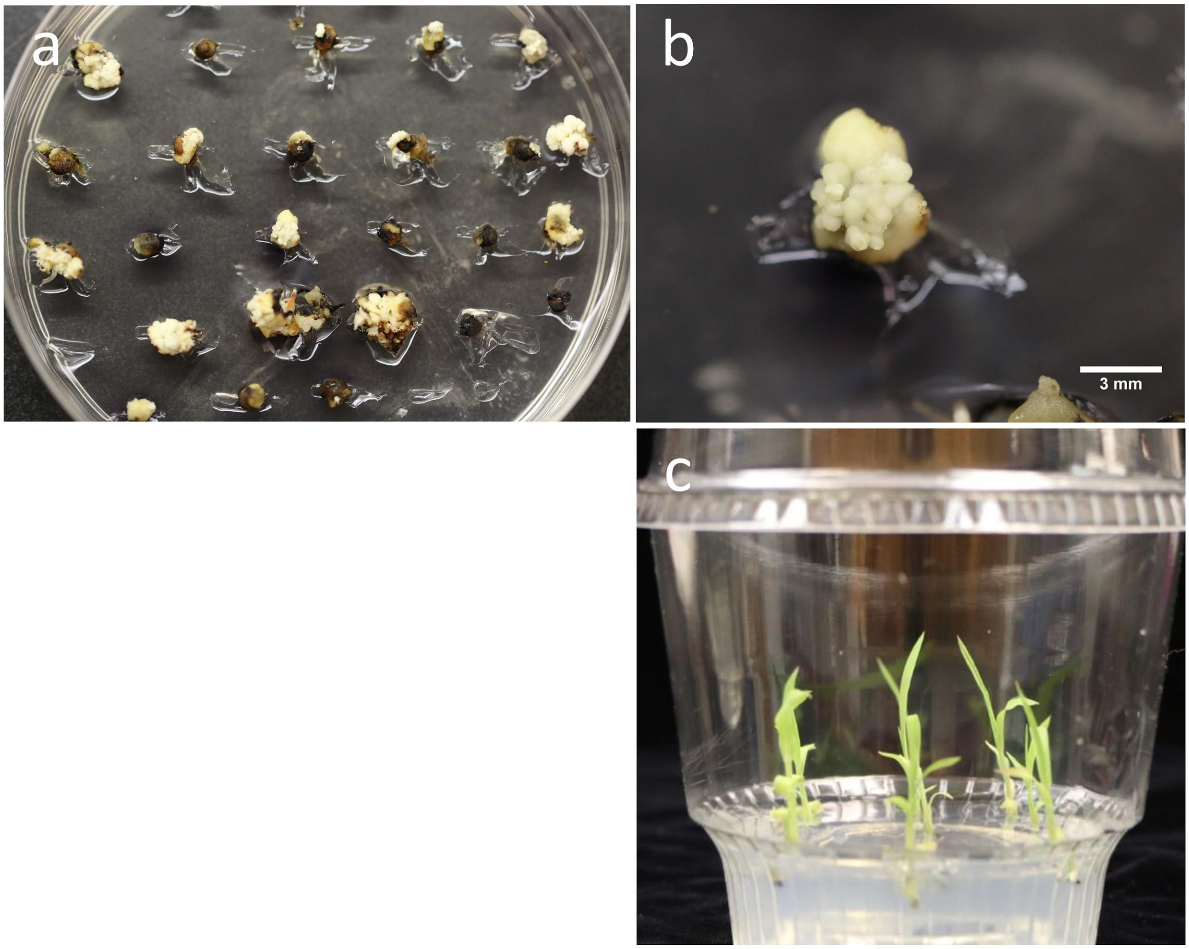

Figure S1 (a, b) BTx642 tissues on EMM with IMZ selection following transformation with pPHP81814. (c) Plantlets growing on rooting media with IMZ.

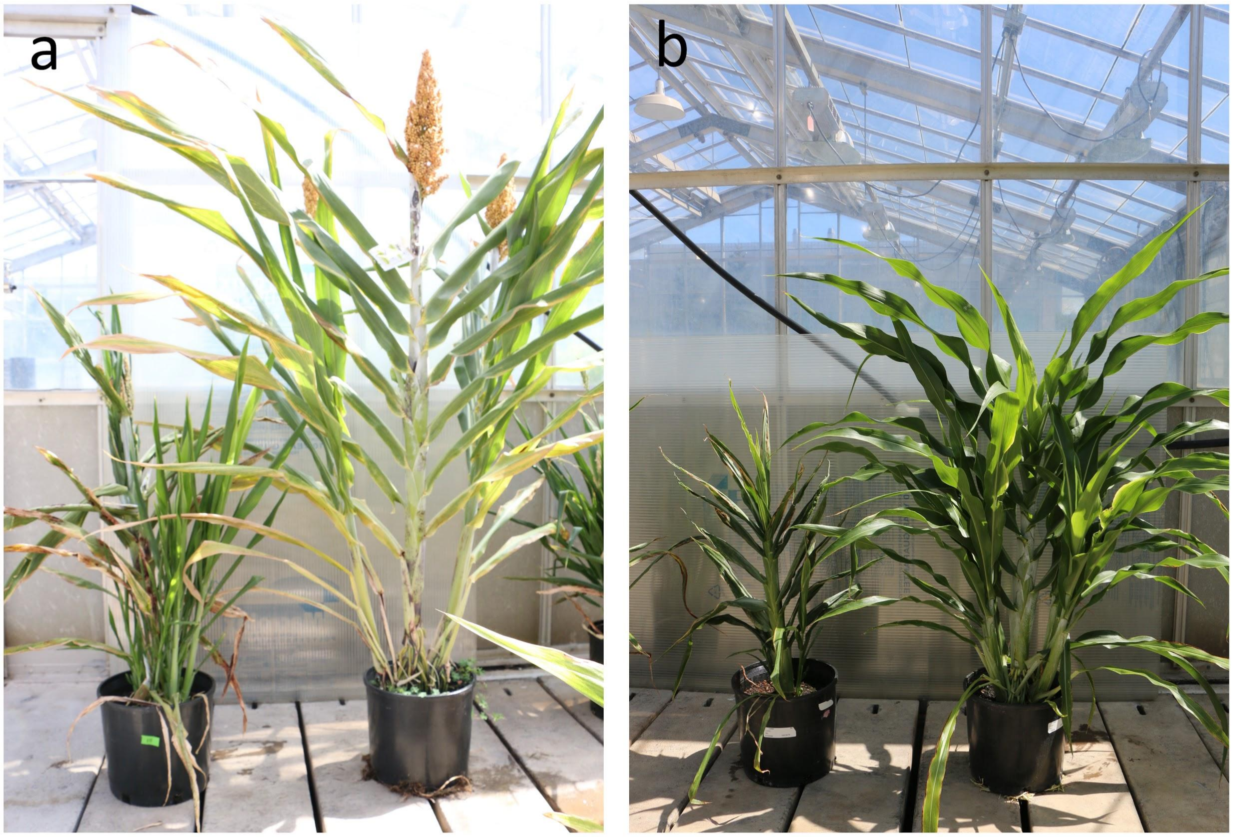

Figure S2 Plants from (a) RTx430 and (b) BTx642 transformed with pPHP81814. In both, plants null for *Zm-Bbm* and *Wus2* (right) and plants retaining introduced *Zm-Bbm* and *Wus2* (left) have shorter stature, twisted leaves and poorer seed set, compared to wild-type.

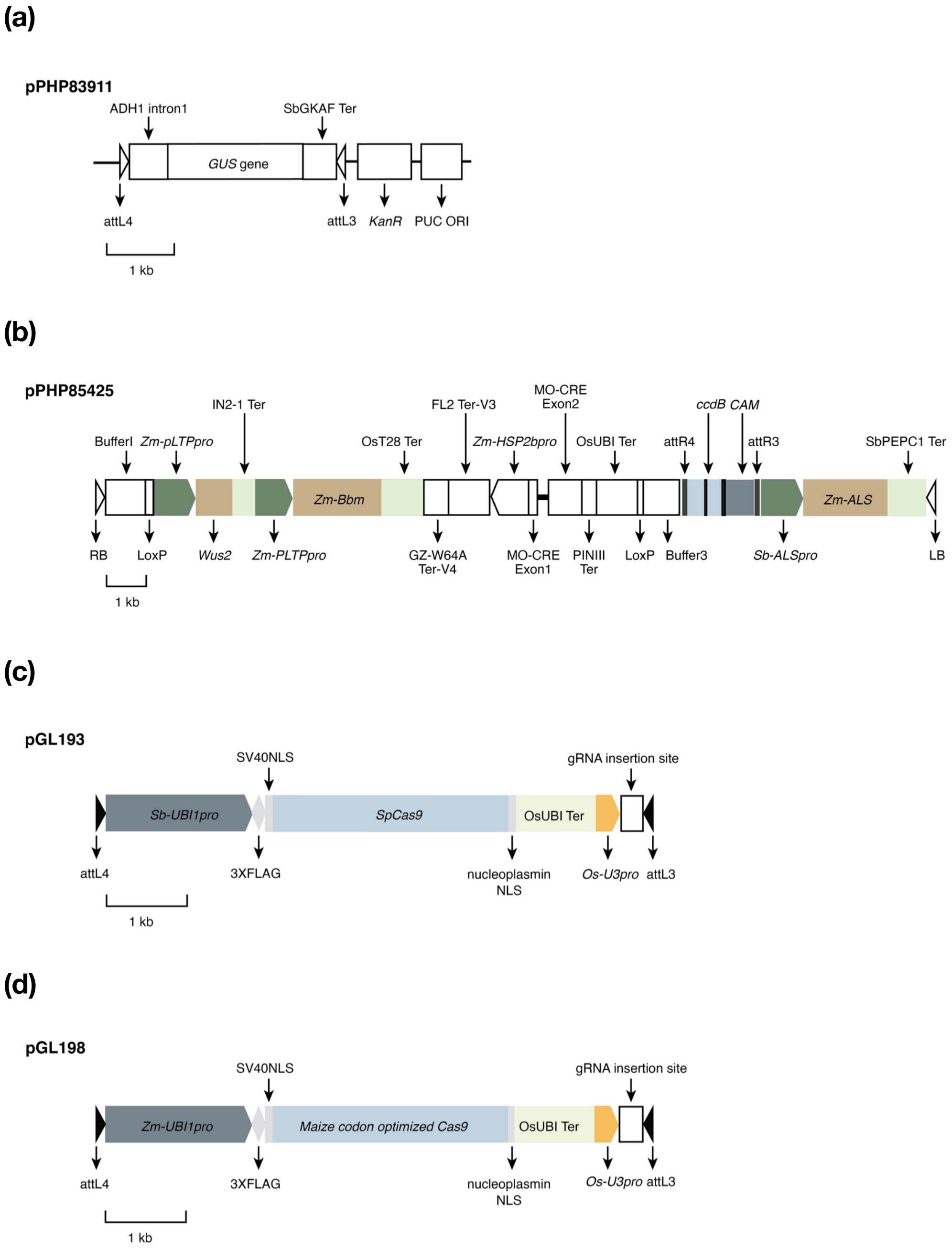

Figure S3 Schematic representation of the molecular components of constructs. (a) pPHP83911, used for generating pGL193 and pGL198, is a pENTR vector. (b) pPHP85425 is a destination vector for morphogene-assisted transformation (MAT). Molecular components between left and right borders are shown. (c) pGL193 is a pENTR vector for MAT-mediated editing with gRNAs. The remaining backbone part is from pPHP83911. (d) pGL198 is a pENTR vector for MAT-mediated editing with the maize ubiquitin promoter and maize codon-optimized SpCas9 for carrying gRNAs. The remaining backbone part is also from pPHP83911.

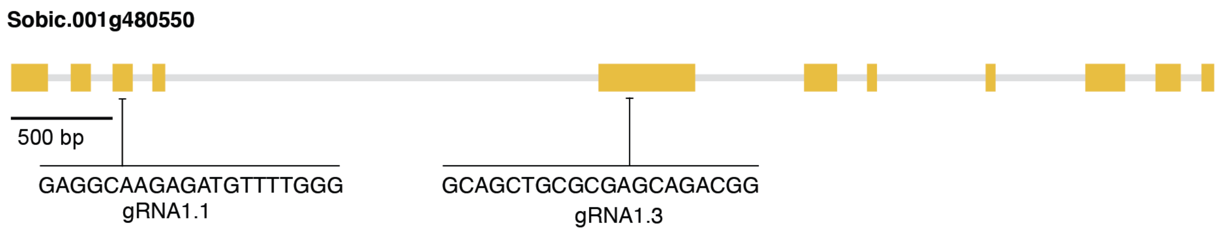

Figure S4 Two gRNAs targeting *pds* in pGL196; yellow rectangles indicate exons; light gray lines indicate intron regions. gRNA1.1 is located in the third exon of *pds* genes. gRNA1.3 is located in the fifth exon of *pds* genes.

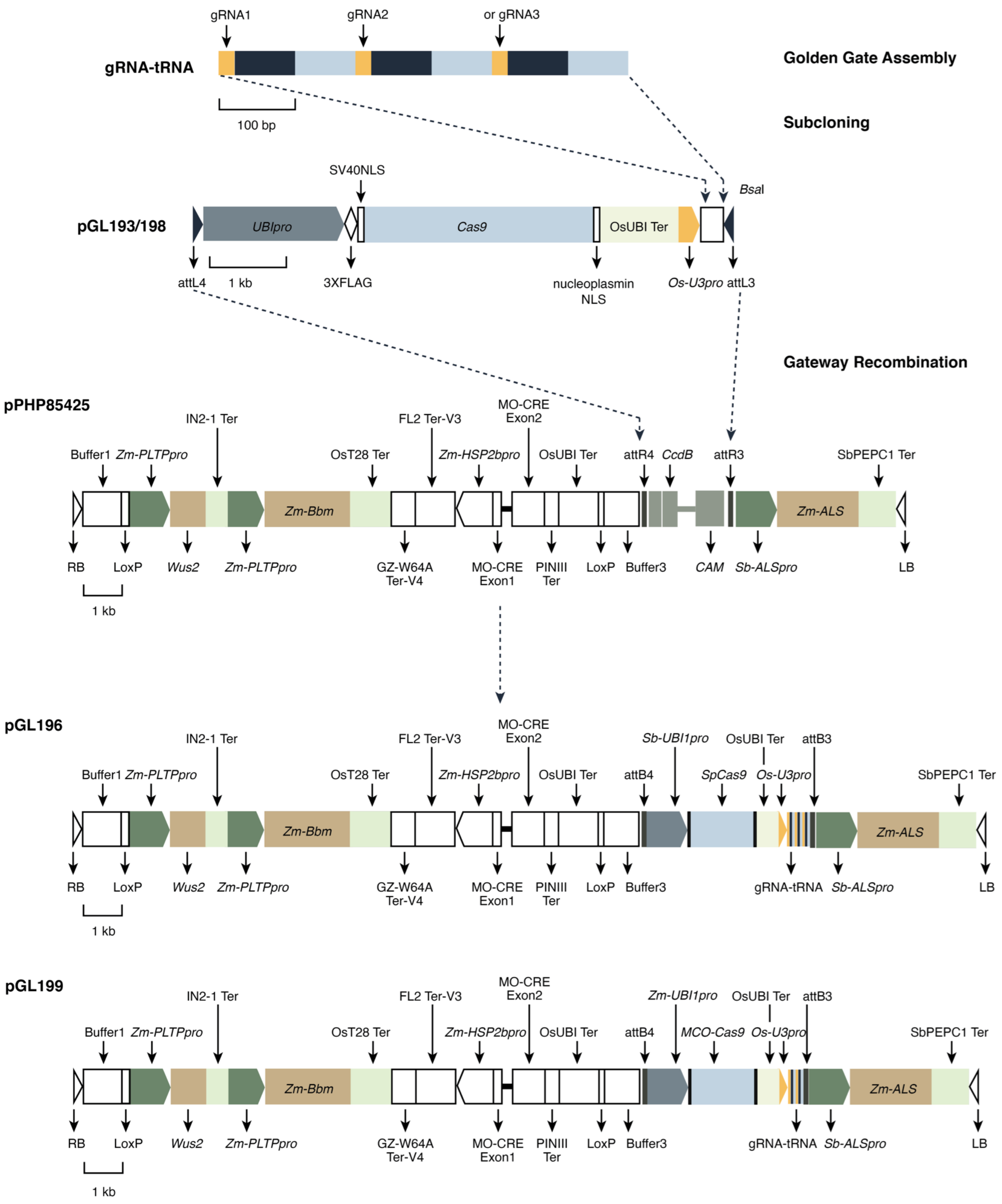

Figure S5 Molecular cloning strategy for constructing sorghum CRISPR/Cas9 vectors. Cloning vector for gRNAs containing *Bsa*I sites for sequential insertion of two or three gRNAs with tRNAs that forms polycistronic cassettes (gRNA-tRNA), driven by rice U3 promoter (*Os-U3pro*). The gRNA-tRNA and Cas9 expression cassettes were mobilized into pPHP85425 through Gateway recombination, generating pGL196 and pGL199 for Agrobacterium-mediated sorghum transformation.

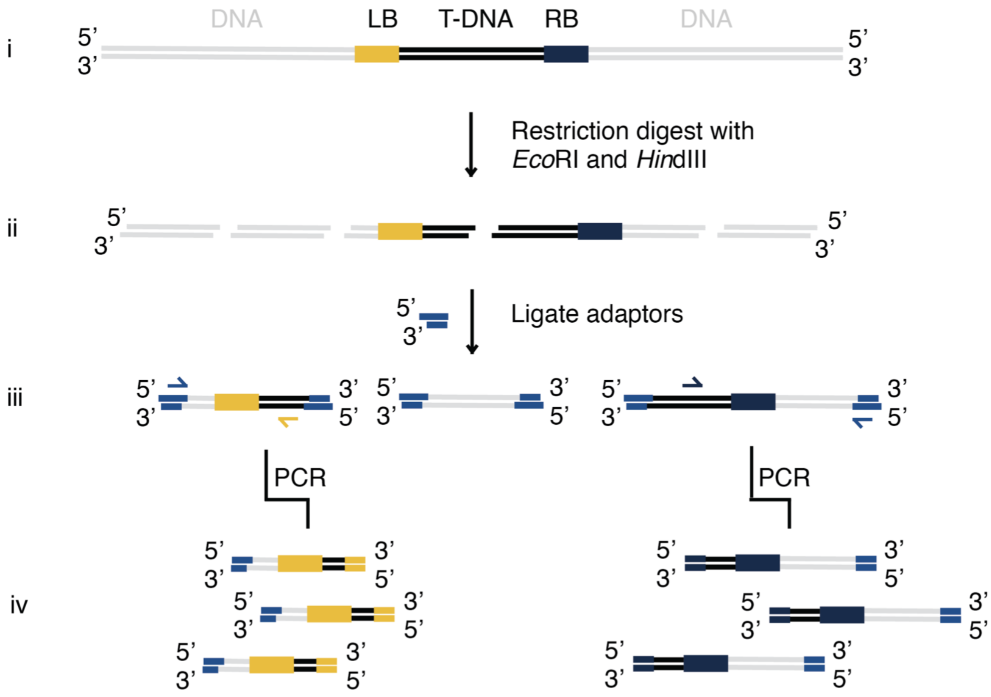

Figure S6 Determination of transgene independent integration (TII) with adapter ligation-mediated PCR**.** Genomic DNA with a T-DNA insert (i) was digested with *Eco*RI and *Hin*dIII (ii)**.** Adapters are ligated to digested sites creating adapter-flanked templates (iii). Only the longer arm of the adapter contains a sequence exactly matching the adapter primers. Adapter-to-adapter amplification does not occur due to lack of primer match sites on the shorter arm of adapters. If T-DNA is present in the template (black line, Fig. 4a.i), T-DNA primers (yellow arrow, left border; dark blue arrow, right border, Fig. 4 a.iii) will bind to their corresponding sites and initiate synthesis of a complementary strand (iv)**.** PCR products will contain adapter primer-binding sites derived from the complement of the longer arm of the adapter.

Table S1. Primers for genotyping putative transformed plants

Gene symbol	Protein name	Construct name	Primers (F/R) (5'-3')	Amplicon length (bp)	Tm (°C)
ALS2	Acetolactate synthase	pPHP81814	CTTTGGCTCATGGAACGA<br>ATCTTCTTTATCGCTGCGC	576	58.2
ZSG	ZsGreen	pPHP81814	CTCCTGCGAGAAGATCATC<br>ACCCTATCGAGATCTGAGTC	277	55
RFP	Red fluorescence protein	pANIC10A	GGCCATTATACGTGCGACTT<br>GCATGTGCAATTTCTCGTTG	155	60
Bbm	Baby boom	190	GCCGGAGCAACCACTACAT<br>TCCGCCACCATTGTTCTC	132	61
hph	Hygromycin phosphotransferase	pANIC10A	GAAGAATCTCGTGCTTTCAGCTTCG<br>CAAGCTCTGATAGAGTTGGTCAAGACC	741	64
Bbm	Baby boom	pGL190/ pGL196/ pGL199	GGTCGTCAAGTCTATTTAGGTGGCT<br>AAGTAAAGATCCTTGTTCCCTGCAACT	299	63
gRNA unit	gRNA-tRNA polycistronic cassette	pGL196/ pGL199	CCTAGAAGGCCACCCAGCGGATAACAATTTCA<br>CTGCAGGCATGCACGCGCTAAAAAC	1,157	60
PDS-06g-1	Phytoene desaturase	pGL196	TGTAAGTTGGGGAATTTCGAGGGAACCACTA<br>GCAGAGTTAAGCTCAACGGTAATGGTGTAGTG	1,127	60
PDS-06g-2	Phytoene desaturase	pGL199	TAATTAATCCATCAATTGTTCTCAGC<br>GGTTGTTAATATGTGTAGAAACCATCA	1,061	60

Table S2. Primers for constructs, gRNAs and determining transgene independent integration (TII)

Accession Name	Purpose	Primer Name	Sequences 5 -> 3
pPL01	pGL190 construction	attL4 R	caacttttctatacaaagttggcattataaaaaagcattgcttatcaat
pPL02	“	attL4_pUBI F	gccaactttgtatagaaaagttgctgcccttaaggccaattgttcaagattcattcaaca
pPL03	“	attL3_tUBI R	ggtgaccggcgcgccgaagcctaccaaagcaaagcgtttgtcga
pPL04	“	attL3 F	ggcgcgccggtcacccggt
pPL05	“	ZS Green 2 R	accctatcgagatctgagtc
pPL06	pGL193 construction	Cas9 F	ctttaacttagcctaggatccgactataaggaccacgacggagactacaaggatca
pPL07	“	Cas9 R	actggccattggtacccttttcttttttgcctggccggcctttttcgtg
pPL08	“	pENTR F	ggtaccaatggccagttaacagatccagct
pPL09	“	pENTR R	ggatcctaggctaagttaaagtcgacctgcag
pPL10	“	gRNA unit F	cctagaaggccacccagcggataacaatttcacacaggaaacagctatgaca
pPL11	“	gRNA unit R	aactttgtataataaagttgcccttgtggacctgcaggcatgcacg
pPL12	gRNAs for sorghum PDS	PDS-gR1.1 F	taGGTCTCCAGATGTTTTGGGgttttagagctagaa †
pPL13	“	PDS-gR1.1 R	cgGGTCTCAATCTCTTGCCTCtgcaccagccggg
pPL14	“	PDS-gR1.2 F	taGGTCTCCGGAGAATTCAGCgttttagagctagaa
pPL15	“	PDS-gR1.2 R	cgGGTCTCACTCCTGGCTTGTtgcaccagccggg
pPL16	“	PDS-gR1.3 F	taGGTCTCCGCGAGCAGACGGgttttagagctagaa
pPL17	“	PDS-gR1.3 R	cgGGTCTCATCGCGCAGCTGCtgcaccagccggg
pPL18	Determining independent event	LSA 1	gtaatacgactcactatagggcacgcgtggtcgacggcccgggctgc
pPL19	“	SSAH3	phosphate-AGCTGCAGCCCG-amino C7 ‡
pPL20	“	SSAE1	phosphate-AATTGCAGCCCG-amino C7

pPL21	"	LBa1	tcacaattccacacaacatacgagcc
pPL22	"	RBa1	gccagctggcgtaatagcgaaga
pPL23	"	AP1	gtaatacgactcactatagggcacgcg
pPL24	"	LBb1	attaattgcgttgcgctcactgccc
pPL25	"	RBb1	cccaacagttgcgcagcctgaatggcgaat
pPL26	"	AP2	tggtcgacggcccgggctgc
pPL27	pGL198 construction	ZM-UBI F	gccaactttgtatagaaaagttgctgcagtgcagcgtgacccg
pPL28	"	ZM-opt Cas9 R	actggccattggtacccttcttcttcttcgcctgccccgc
pPL29	pGL199 construction	PDS-gR2.1 F	taGGTCTCCAACGGTTTACTGgttttagagctagaa
pPL30	"	PDS-gR2.1 R	cgGGTCTCACGTTGCAGGTCCtgcaccagccggg
pPL31	"	PDS-gR2.2 F	taGGTCTCCTTGGTACGAGACgttttagagctagaa
pPL32	"	PDS-gR2.2 R	cgGGTCTCACCAATCCCCATCtgcaccagccggg
pPL33	Transgene independent insertion (TII) determination for altruistic MAT §	LBa1_81814	ccgattaatcgtggcctcttgct
pPL34	"	LBb1_81814	cgtggcctcttgctcttcaggatgaagagctatgt

† Capitalized letters indicate gRNA sequences.
‡ Capitalized letters indicate oligonucleotides.
§ MAT

Table S3. Medium composition for sorghum transformation

Medium name	Medium components
PHI-I (infection medium)	MS salts plus vitamins, 4.43 g/L, thiamine-HCl 1mg/l, 2,4-D 1.5 mg/l, sucrose 68.5 g/l, glucose 36 g/l, acetosyringone[1] 39.24 mg/l, pH 5.2.
Co-cultivation	MS salts plus vitamins, 4.43 g/L, thiamine-HCl 1mg/l, 2,4-D 2 mg/l, sucrose 20 g/l, glucose 10 g/l, L-proline 0.7 g/l, MES buffer 0.5 g/l, acetosyringone[†] 39.24 mg/l, ascorbic acid[†] 10 mg/l, thymidine[†] 100mg/l, pH 5.8.
Resting	MS salts plus vitamins, 4.43 g/L, thiamine-HCl 1 mg/l, 2,4-D 2 mg/l, sucrose 20 g/l, glucose 10 g/l, L-proline 0.7 g/l, MES buffer 0.5 g/l, acetosyringone[†] 39.24 mg/l, ascorbic acid[†] 10 mg/l, carbenicillin[†] 25 mg/l, pH 5.8.
[‡] Embryo-maturation medium (EMM)	MS salts plus vitamins, 4.43 g/L, zeatin 0.05 mg/l, copper sulfate 1.25mg/l, L-proline 0.7 g/l, sucrose 60 g/l, IAA[†] 1 mg/l , ABA[†] 0.026mg/l , thidiazuron 0.1 mg/l, BAP[†] 1 mg/l, carbenicillin[†] 250mg/l, pH 5.6.
[‡] Rooting (RM)	MS salts 4.43 g/l, sucrose 40 g/l, pH 5.6

[†] Added after medium is autoclaved and cooled to ~55℃

[‡] For EMM and RM, selection agent (hyg or IMZ) differs depending on construct

Table S4. Constructs used for sorghum transformation

Construct	Construct Size	Purpose	Plant selectable marker gene (selection agent)	Bacterial selectable marker gene (selection agent)
pPHP81814	27.8 kb	MAT †	Acetolactate synthase, *ALS* (Imazapyr)	Adenylyltransferease, *aadA* (spectinomycin)
pANIC10A	17.4 kb	Altruistic MAT	hygromycin phosphotransferase, *hph* (hygromycin)	Aminoglycoside phosphotransferase, *nptIi*, (kanamycin)
pGL190	31.4 kb	Altruistic MAT	Acetolactate synthase, *ALS* (Imazapyr)	Adenylyltransferase, *aadA* (spectinomycin)
pGL193	10.3 kb	Entry vector for gRNAs targeting *pds*	NA	Aminoglycoside phosphotransferase, *nptII,* (kanamycin)
pGL196	36.1 kb	MAT-mediated CRISPR editing target for *Pds*	Acetolactate synthase, *ALS*	Adenylyltransferase, *aadA* (spectinomycin)
pGL198	10.4 kb	Entry vector	NA	Aminoglycoside phosphotransferase, *nptII,* (kanamycin)
pGL199	36.1 kb	MAT-mediated CRISPR editing target for *Pds*	Acetolactate synthase, *ALS*	Adenylyltransferase, *aadA* (spectinomycin)

† MAT- morphogene-assisted transformation

Table S5. PCR programs for genotyping putative transformed plants

Gene Symbol	Gene Name	Construct name	Initial Denature	Denature	Annealing	Extension	Extension	Hold	Polymerase
ALS2	Acetolactate synthase	pPHP81814	95°C-3 min	95°C-30 sec	54°C-30 sec	72°C-60 sec	72°C-5 min	12°C- ∞	EconoTaq ® PLUS GREEN 2X Master Mix
ZSG-1	ZsGreen	pPHP81814	95°C-4 min	95°C-30 sec	55°C-30 sec	72°C-50 sec	72°C-7 min	12°C- ∞	
RFP	Red fluorescence protein	pANIC10A	95°C-3 min	95°C-30 sec	53°C-30 sec	72°C-30 sec	72°C-5 min	4°C- ∞	
Bbm	Baby Boom	pGL190	95°C-3 min	95°C-30 sec	53°C-30 sec	72°C-30 sec	72°C-5 min	4°C- ∞	
hph + *Bbm*	Hygromycin phospho-transferase + Baby Boom	pGL190 + pANIC10A	95°C-3 min	95°C-30 sec	57°C-30 sec	72°C-60 sec	72°C-5 min	4°C- ∞	
gRNA unit	gRNA-tRNA	pGL196	98°C-3 min	98°C-10 sec	60°C-15 sec	68°C-75 sec	68°C-5 min	4°C- ∞	PrimeSTAR GXL
Pds	Phytoene desaturase	pGL199	98°C-3 min	98°C-10 sec	60°C-15 sec	68°C-5 sec	68°C-5 min	4°C- ∞	

Table S6. Primers and probes for digital droplet PCR

Gene symbol	Protein name	Primers (F/R) (5'-3')	Probe sequence (5'-3')	Amplicon length (bp)	Tm (°C)
ALS2†	Acetolactate synthase	CTTTGGCTCATGGAACGA<br>CTGCCCAACACCTGTGC	CATATTGTGGCTGGATCTCCTCA TTAGAT	154	59
RFP†	Red fluorescence protein	CGATGGCGACTCTTTCATCT<br>CGTATAATGGCCACCGTTCT	TGCCACCCACCACACTCATAC	184	60
PP2A‡	Serine/ threonine protein phosphatase	CCGATCTGTGATATGGGACG<br>CTAGACCCAAAAGCAACCCA	TGGTTGGTTTTTGTGCGTCTGG CCGG	209	60

† *ALS* and *RFP* probes: labeled with FAM™, double-quenched with ZEN™ and Iowa Black Hole Quencher®
‡ *PP2A*: labeled with HEX™, double-quenched with ZEN™ and Iowa Black Hole Quencher®

Table S7. Description and references for construct elements used for transformation

Symbol	Name	Reference
Wus2	*Zea mays Wuschel2*	(Lowe et al. 2007)
Zm-Bbm	*Zea mays Baby Boom*	(Gordon-Kamm, W.J., Helentjaris, T.G., Lowe, K.S., Shen, B., Tarczynski, M.C., Zheng, P., n.d.) et al. 2005)
Zs-GREEN1	Green fluorescence protein, *Zoanthus sp*.	(Matz et al. 1999)
moCRE	*Zea mays*-optimized CRE recombinase	(Odell et al. 1990)
loxP	Recombinase target site for CRE recombinase	(Odell et al. 1990)
Sb-ALSpro	*Sorghum bicolor* ALS promoter	SB-ALS promoter and 5’UTR, DOE-JGI Sbi v3.1, SBChr04, bases 49239164-49240031.
Zm-Axig1pro	*Zea mays* auxin-inducible promoter 1	(Garnaat, Lowe, and Roth 2005), NCBI accession AR883375.1 bases 1-1240
Zm-PLTPpro	*Zea mays* phospholipid transferase promoter	GenBank sequence MN380778
Sb-Ubi1pro	*Sorghum bicolor* ubiquitin promoter	Unpublished Corteva Agriscience sequence
Zm-Glb1pro	*Zea mays* globulin 1 promoter	(Liu et al. 1998)
Zm-Hsp26pro	*Zea mays* heat shock promoter	Unpublished Corteva Agriscience sequence
Zm-Ubi1pro	*Zea mays* ubiquitin promoter	(Xing et al. 2014)
